# Maternal consumption of fish oil programs reduced adiposity in broiler chicks

**DOI:** 10.1038/s41598-017-13519-5

**Published:** 2017-10-13

**Authors:** Ronique C. Beckford, Sarah J. Howard, Suchita Das, Abigail T. Farmer, Shawn R. Campagna, Jiali Yu, Robert L. Hettich, Jeanna L. Wilson, Brynn H. Voy

**Affiliations:** 1 Department of Animal Science, University of Tennessee, Knoxville, TN United States; 2 Department of Chemistry, University of Tennessee, Knoxville, TN United States; 3 Graduate School of Genome Science and Technology, University of Tennessee, Knoxville, TN United States; 4 Chemical Sciences Division, Oak Ridge National Laboratory, Oak Ridge, TN United States; 5 Department of Poultry Science, University of Georgia, Athens, GA United States

## Abstract

Maternal intake of eicosapentaenoic acid (EPA; 20:5 n-3) and docosahexaenoic acid (22:6 n-3) has been associated with reduced adiposity in children, suggesting the possibility to program adipose development through dietary fatty acids before birth. This study determined if enriching the maternal diet in fish oil, the primary source of EPA and DHA, affected adipose development in offspring. Broiler chickens were used because they are obesity-prone, and because fatty acids provided to the embryo can be manipulated through the hen diet. Hens were fed diets supplemented (2.8% wt:wt) with corn oil (CO; n-6) or fish oil (FO; n-3) for 28 d. Chicks from both maternal diet groups were fed the same diet after hatch. Maternal FO consumption enriched chick adipose tissue in EPA and DHA and reduced adiposity by promoting more, but smaller, adipocytes. This adipocyte profile was paralleled by lower expression of the adipogenic regulator *PPARG* and its co-activator PPARGC1B, and elevated expression of *LPL*. Proteomics identified 95 differentially abundant proteins between FO and CO adipose tissue, including components of glucose metabolism, lipid droplet trafficking, and cytoskeletal organization. These results demonstrate that the maternal dietary fatty acid profile programs offspring adipose development.

## Introduction

Active proliferation and differentiation of preadipocytes shortly before birth and in the first few years of life creates a sensitive window for adipose development^1–3^. Consequently, the maternal diet and in utero environment can impact adipose deposition and the consequent risk for obesity. Adipose tissue is subject to developmental programming, in which exposures in utero exert lasting effects on tissue phenotypes. Variations in the maternal diet, lifestyle and environmental exposures have been linked to increased adiposity later in life through stable effects on adipocyte growth and metabolism^4–6^. Programming of adipose tissue is of particular public health interest because obesity, which is epidemic in the U.S. and globally, begins early in life. Approximately 27% of children in the U.S. are overweight or obese by age five^7^. Obese children are much more likely to be obese as adults when compared to normal weight children^8,9^. Therefore, limiting excess fat accumulation in the first few years of life is an important tool for the prevention of adult obesity. Evidence that adiposity at birth predicts fatness later in childhood highlights the need to understand prenatal factors that influence adipose development^10,11^.

The types of fatty acids provided in the maternal diet may influence early adipose development and the resultant propensity for fat accumulation in children. *In vitro* studies with adipocyte cell lines demonstrate that polyunsaturated fatty acids (PUFAs) of the n-3 and n-6 series differentially regulate preadipocyte proliferation, adipogenesis, and triglyceride storage. Omega-6 PUFAs, particularly arachidonic acid (AA; 20:4 n-6) tend to be pro-adipogenic^12–14^, while LC n-3 PUFAs (e.g., eicosapentaenoic acid (EPA; 20:5 n-3) and docosahexaenoic acid (DHA; 22:6 n-3) attenuate lipid accumulation and promote an oxidative adipocyte phenotype^15–17^. Fatty acid profiles of diets in the US and other industrialized countries have shifted over the last several decades to favor consumption of n-6 PUFAs at the expense of n-3 PUFAs^18^. Both fatty acids supplied to the developing embryo and the fatty acid profiles of breast milk directly reflect the maternal diet^19^, creating the potential to impact the earliest stages of adipose development. Epidemiological attempts to associate maternal dietary fatty acid profiles with fat mass in children have been inconclusive. Two recent prospective studies demonstrated an inverse relationship between levels of n-3 PUFAs in maternal blood during pregnancy and fatness in childhood^20,21^. However, relative contributions of the pre- and perinatal maternal diet are difficult to separate from shared consumption patterns after lactation in human studies.

Avians provide a unique model in which to specifically manipulate the pool of fatty acids that are supplied to the embryo and test the effects on adipose deposition after hatch (i.e. birth). The yolk provides the majority of fatty acids to developing tissues in the embryo, and for one to two days after hatch, until feeding is established. The fatty acid profile of the yolk can be modified through the source of dietary fat provided to the hen^22,23^. For example, commercial eggs that are enriched in EPA and DHA are produced by supplementing the hen’s diet with marine oils. We used this relationship to test the hypothesis that enriching the embryo in EPA and DHA, supplied in fish oil (FO), reduces adipose deposition in chicks. Corn oil (CO) was used as a reference because it contains a comparable level of PUFA (~ 60%), but primarily those of the n-6 family. All chicks were fed a CO-based diet after hatch to confine the experimental manipulation to the period of embryonic development. We demonstrate that maternal FO feeding significantly reduced adiposity after hatch, with no effect on growth. Our results suggest that fatty acids in the maternal diet contribute to developmental programming of adipose tissue.

## Results

### Egg production and chick tissue fatty acid composition

Hatchability, egg weights, and chick weight at hatch were used to assess the effect of hen diet on egg quality, none of which differed significantly between eggs from CO and FO hens (*P* > 0.05). The fatty acid compositions of phospholipids contained in brain and liver were profiled and qualitatively compared to confirm that EPA and DHA were enriched in tissues of FO chicks, compared to CO chicks, at hatch. Brain and liver were used because of their relative mass at hatch. Enrichment levels of phosphatidylcholine (PC) species containing EPA and DHA are shown in Table 1. The fold-increase (FO/CO) ranged from ~ 1.2 (18:0/22:6 in brain) to ~ 130.4 (PC 18:4/22:6 in liver), with greater than 2-fold enrichment for most species. These data confirm that the maternal dietary fatty acid profile was reflected in tissue fatty acid composition of the resultant chicks at the time of hatch.

**Table 1:** Enrichment of brain and liver phospholipids at hatch in broiler chicks produced from hens fed diets containing CO or FO for 28 d.

PC species	FO/CO ratio	
	Brain	Liver
PC (38:10)	7.40	130
PC (36:5)	13.9	13.9
PC (34:5)	21.5	11.1
PC (42:11)	11.4	6.62
PC (34:6)	4.98	4.79
PC (38:8)	6.29	4.76
PC (44:12)	1.65	4.01
PC (40:6)	1.19	2.51
PC (38:7)	2.16	2.43
PC (36:6)	1.85	2.09
PC (40:9)	2.41	1.71

FO/CO ratio was calculated from the relative abundance of each species in brain and liver to qualitatively evaluate tissue enrichment immediately after hatch, using N = 2 per tissue and diet group; CO, corn oil; FO, fish oil.

The effect of maternal fatty acid source on the fatty acid composition of developing adipose tissue was quantified using GC-FID. Fatty acid composition of the total lipid fraction of abdominal adipose tissue was analyzed at 7 and 14 d of age (Table 2). Tissue abundance of five fatty acids (palmitoleic, γ-linolenic, eicosenoic, eicosadienoic and docosadienoic acids) increased with age (*P*_Age_ < 0.05) but was not affected by diet or diet X age interactions. Adipose tissue of FO chicks was significantly enriched in EPA and DHA (*P*_Diet_ < 0.01). At 7 d, tissue from FO chicks contained approximately six times more EPA than tissue from CO chicks. Enrichment declined from 7 to 14 d as all chicks consumed a CO-based diet (*P*_Age*Diet_ < 0.01), but still differed by ~ 2-fold. Comparable effects were seen for DHA content, which was increased by ~ 2.5-fold at 7 d and ~ 1.8-fold at 14 d. As for EPA, the relative enrichment declined with age (*P*_Age*Diet_ = 0.03), and did not differ significantly at 14 d. Fatty acid composition of PC species in abdominal adipose tissue at 7 d also reflected the maternal diet. Six species containing EPA or DHA were significantly increased, by 3- to 4-fold, in FO vs. CO tissue (Supplemental Table 1).

**Table 2:** Abdominal adipose tissue fatty acid content of broiler chicks produced from hens fed diets containing CO or FO for 28 d.

Fatty acid (mol %)	CO		FO				*P*-value	
	7 d	14 d	7 d	14 d	SEM	Diet	Age	Diet × Age
Capric, 10:0	0.007	0.007	0.005	0.009	0.001	0.28	0.45	0.28
Lauric, 12:0	0.024	0.020	0.024	0.027	0.002	0.13	0.81	0.13
Myristic, 14:0	0.520	0.462	0.555	0.577	0.031	0.06	0.70	0.21
Myristoleic, 14:1	0.144	0.154	0.152	0.195	0.015	0.13	0.11	0.31
Pentadecanoic, 15:0	0.058	0.056	0.060	0.054	0.007	0.10	0.61	0.77
Palmitic, 16:0	22.4	22.1	23.4	28.9	1.92	0.61	0.20	0.15
Palmitoleic, 16:1	5.17	6.09	5.61	7.94	0.610	0.08	0.01	0.27
Heptadecanoic, 17:0	0.096	0.081	0.095	0.085	0.006	0.94	0.33	0.83
Stearic, 18:0	5.72	5.03	5.43	6.38	0.509	0.49	0.87	0.29
Oleic, 18:1	30.7	30.0	31.2	18.2	4.36	0.21	0.14	0.17
Linoleic, 18:2n-6 *cis*	16.5	17.2	15.9	18.0	1.37	0.99	0.39	0.55
Linoleladic,18:2n-6 *trans*	16.5	16.8	15.6	17.6	1.78	0.99	0.39	0.55
γ-Linolenic 18:3n-6	0.180	0.206	0.164	0.209	0.015	0.68	0.03	0.55
α-Linolenic, 18:3n-3	0.718	0.672	0.689	0.722	0.067	0.88	0.92	0.56
Arachidic, 20:0	0.052	0.056	0.052	0.071	0.006	0.26	0.11	0.30
Eicosenoic, 20:1n-9	0.289	0.367	0.280	0.439	0.027	0.27	<0.01	0.16
Eicosadienoic, 20:2n-6	0.134	0.155	0.137	0.169	0.010	0.45	0.03	0.60
Eicosatrienoic, 20:3n-3	0.131	0.142	0.143	0.166	0.001	0.12	0.15	0.60
Dihomo- γ-Linolenic, 20:3n-6	0.205	0.189	0.145	0.145	0.001	0.07	0.78	0.77
Eicosapentaenoic, 20:5n-3	0.009^b^	0.010^b^	0.060^a^	0.02^a^	0.005	<0.01	<0.01	<0.01
Arachidonic, 20:4n-6	0.014	0.012	0.009	0.009	0.001	0.69	0.06	0.53
Erucic, 22:1n-9	0.015	0.013	0.014	0.016	0.001	0.29	1.00	0.20
Docosadienoic, 22:2n-6	0.031	0.037	0.031	0.043	0.003	0.29	<0.01	0.36
Tricosanoic, 23:0	0.050	0.035	0.030	0.029	0.006	0.045	0.20	0.25
Docosahexaenoic, 22:6n-3	0.036^b^	0.016^b^	0.092^a^	0.028^b^	0.007	<0.01	<0.01	0.03
Nervonic, 24:1n-9	0.017	0.006	0.008	0.008	0.004	0.39	0.17	0.20
Activity index^3^								
SCD-16	0.226	0.286	0.240	0.282	0.028	0.85	0.08	0.75
SCD-18	5.41	7.47	5.90	3.68	1.32	0.23	0.95	0.12
D6D	0.006	0.01	0.004	0.010	0.002	0.57	0.01	0.57
DNL	0.683	0.646	0.753	0.084	0.132	0.11	0.91	0.50
EL	0.260	0.220	0.232	0.216	0.019	0.40	0.15	0.53

Fatty acid content of abdominal adipose tissue, represented as mol%. Values are means from N = 5 per diet and age group (7 d and 14 d). SEM values are the pooled SEM across groups; CO, corn oil; FO, fish oil. P-values are from ANOVA F-tests for main effects of diet, age and their interaction (diet × age). Labeled means are from post-hoc comparisons using LSM, calculated following a significant ANOVA F-test for diet × age interaction. Those in a row without a common letter differ; *P* < 0.05. Indices of enzyme activities were calculated from mol%, using the following equations: SCD (Stearoyl-CoA desaturase)-16 (16:1n-7/16:0); SCD-18 (18:1n-9/18:0); D6D (Delta-6 desaturase) (18:3n-6/18:2n-6); DNL (De novo lipogenesis) (16:0/18:2n-6); EL (Elongation) (18:0/16).

### Body weight and adipose deposition

**Figure 1:**
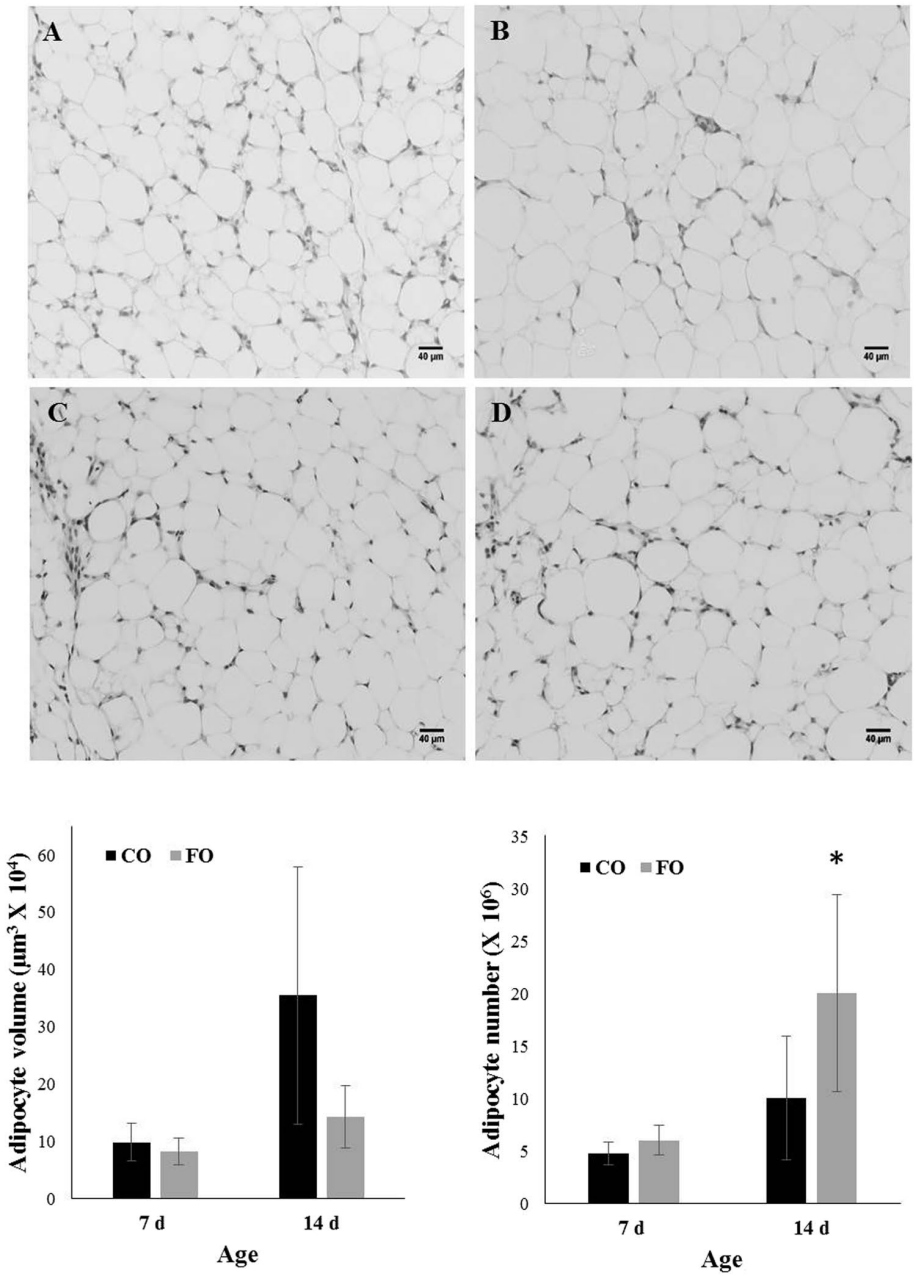
Adipocyte volume and number in CO and FO chicks at 7 and 14 d of age. Representative H&E-stained images of abdominal adipose tissue from CO (**A**,**B**) and FO (**C**,**D**) used to determine adipocyte volume at 7 (**A**,**C**) and 14 (**B**,**D**) d are shown. Scale bar = 40 μm. Two slides, and three independent fields per slide, were counted in three chicks in each age/diet group. (**E**) Average adipocyte volume (μm^3^ × 10^4^), ±SD; (**F**) average adipocyte number (X 10^6^), ±SD. Main effects of diet, age and their interaction (diet × age) on adipocyte volume and number were determined by ANOVA. Significant F-tests (*P* < 0.05) for diet × age interaction were followed by post-hoc comparisons made using LSM. *Different from CO, *P* < 0.05; CO, corn oil; FO, fish oil.

**Figure 2:**
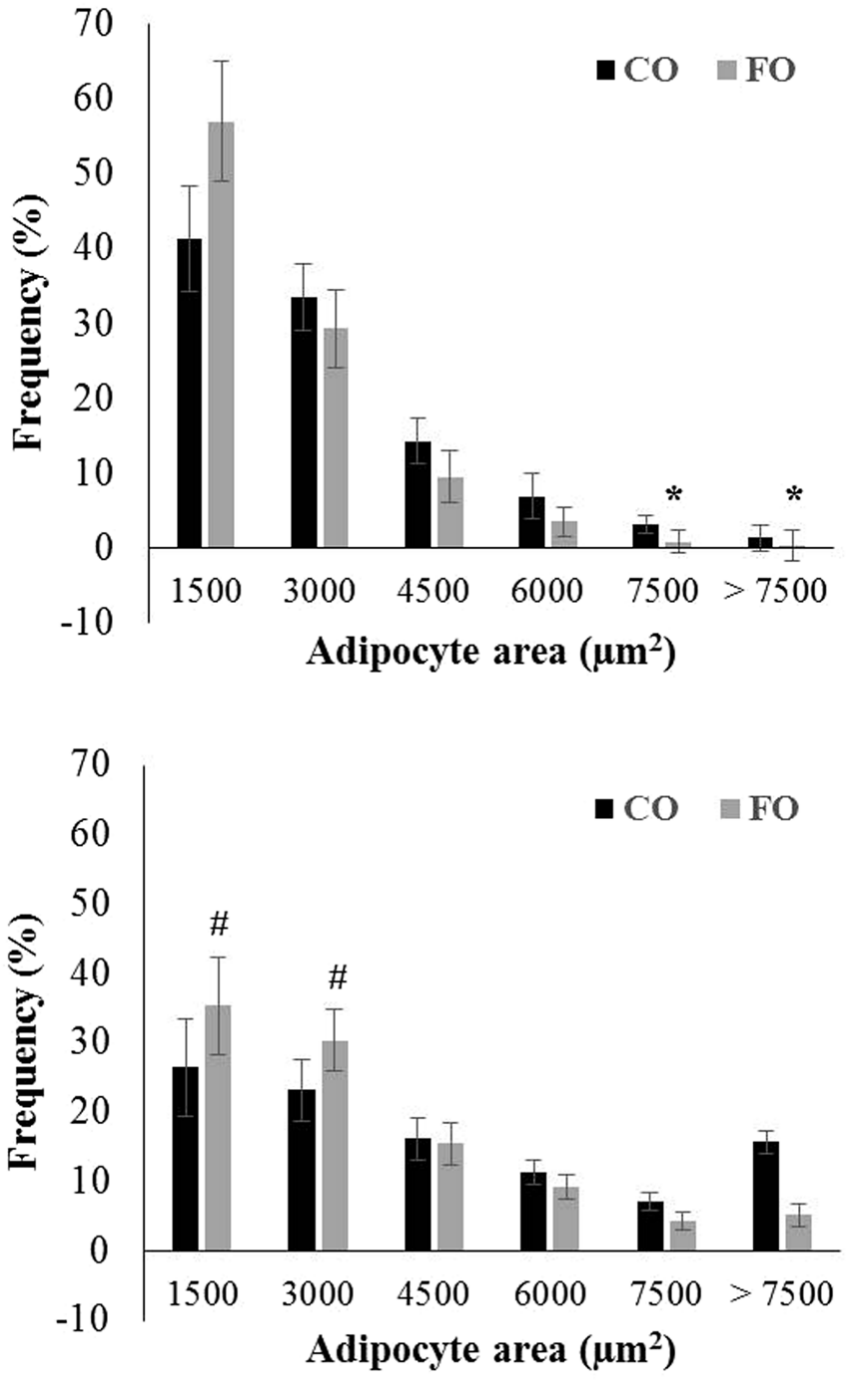
Frequency distributions of adipocyte area (μm^2^) of CO and FO chicks at 7 (**A**) and 14 (**B**) d of age. Adipocyte areas were measured from H&E-stained images and divided into bins by size. Average frequencies of cells within each bin, ± SD, are shown and were compared by T-test. *Different from CO, *P* < 0.05; ^#^different from CO, *P* < 0.10, FO vs. CO; CO, corn oil; FO, fish oil.

Subcutaneous adipose tissue in chickens develops in the embryo and is visible at hatch, while the abdominal depot develops in the first few days after hatch. Weights of both depots were measured to assess the effect of FO in the maternal diet on offspring fat deposition. Body weights did not differ significantly between CO and FO chicks at either 7 or 14 d (Table 3). Food intake did not differ between CO and FO chicks (data not shown). Maternal fatty acid source regulated adiposity of both depots in an age-specific manner. At 7 d, subcutaneous and abdominal adiposity did not differ significantly between FO and CO chicks (*P* > 0.05). However, relative weights of both depots were significantly reduced in FO vs. CO chicks (*P* < 0.05) at 14 d. On average, FO reduced adiposity by ~ 38% in each of the two depots. Effects of maternal diet on adiposity were not associated with diet-induced differences in glycemia or lipolysis, as plasma glucose and NEFA levels were comparable between treatments at both ages (Table 3). The effects of maternal FO on abdominal adipose tissue were further explored because this is the depot in which broilers primarily deposit excess fat. Adipocyte size was measured in H&E-stained sections of abdominal adipose tissue to determine if fatness between groups differed due to cellular hypertrophy (Fig. 1). As expected, adipocyte size increased from 7 to 14 d, reflecting the rapid increase in fat deposition (Fig. 1E). Average cell size did not differ between FO and CO at 7 d (*P* = 0.21) but was significantly reduced in FO at 14 d (*P* = 0.03). Adipocyte number (Fig. 1F), calculated based on adipocyte volume and adipose mass, showed a corresponding significant increase in FO vs. CO chicks at 14 (*P = *0.03) but not 7 d of age (*P = *0.12). Analysis of the adipocyte size distribution revealed that FO favored the abundance of small adipocytes, while there was a greater frequency of larger adipocytes in the CO chicks (Fig. 2). At 7 d there was a significantly lower percentage of adipocytes in three of the larger bin sizes in the FO chicks (*P* < 0.05; Fig. 2A). This effect persisted at 14 d with approximately a 3-fold difference between FO and CO in the percentage of adipocytes (*P* = 0.02) in the largest bin (Fig. 2B). Conversely, approximately 66% of FO adipocytes were in the two smallest bins, compared to 49% in CO.

**Table 3:** Effect of dietary enrichment on performance and serum metabolites of broiler chicks produced from hens fed diets containing CO or FO for 28 d.

	CO		FO			*P*-value		
	7 d	14 d	7 d	14 d	SEM	Diet	Age	Diet × Age
Hatch body wt (g)	41.6	41.6	40.6	38.5	1.04	0.06	0.31	0.33
Final body wt (g)	123^b^	241^a^	111^b^	303^a^	26.7	0.35	< 0.01	0.18
Adiposity (%)								
Abdominal	0.34^c^	1.21^a^	0.31^c^	0.75^b^	0.11	0.04	< 0.01	0.06
Subcutaneous	0.91^ab^	1.23^a^	0.85^b^	0.74^b^	0.8	0.02	0.38	0.08
Glucose (mg/dL)	276	303	346	314	92.3	0.29	0.56	0.20
NEFA (mM)	0.57	0.65	0.61	0.64	0.27	0.85	0.49	0.80

Values are means from N = 10 per diet and age group (7 d and 14 d). SEM values are the pooled SEM across groups; CO, corn oil; FO, fish oil. *P*-values are from ANOVA F-tests for main effects of diet, age and their interaction (diet x age). Labeled means are from post-hoc comparisons using LSM, calculated following a significant ANOVA F-test. Labeled means in a row without a common letter differ; *P* < 0.05. Adiposity = (depot weight/body weight) × 100

### QPCR

**Figure 3:**
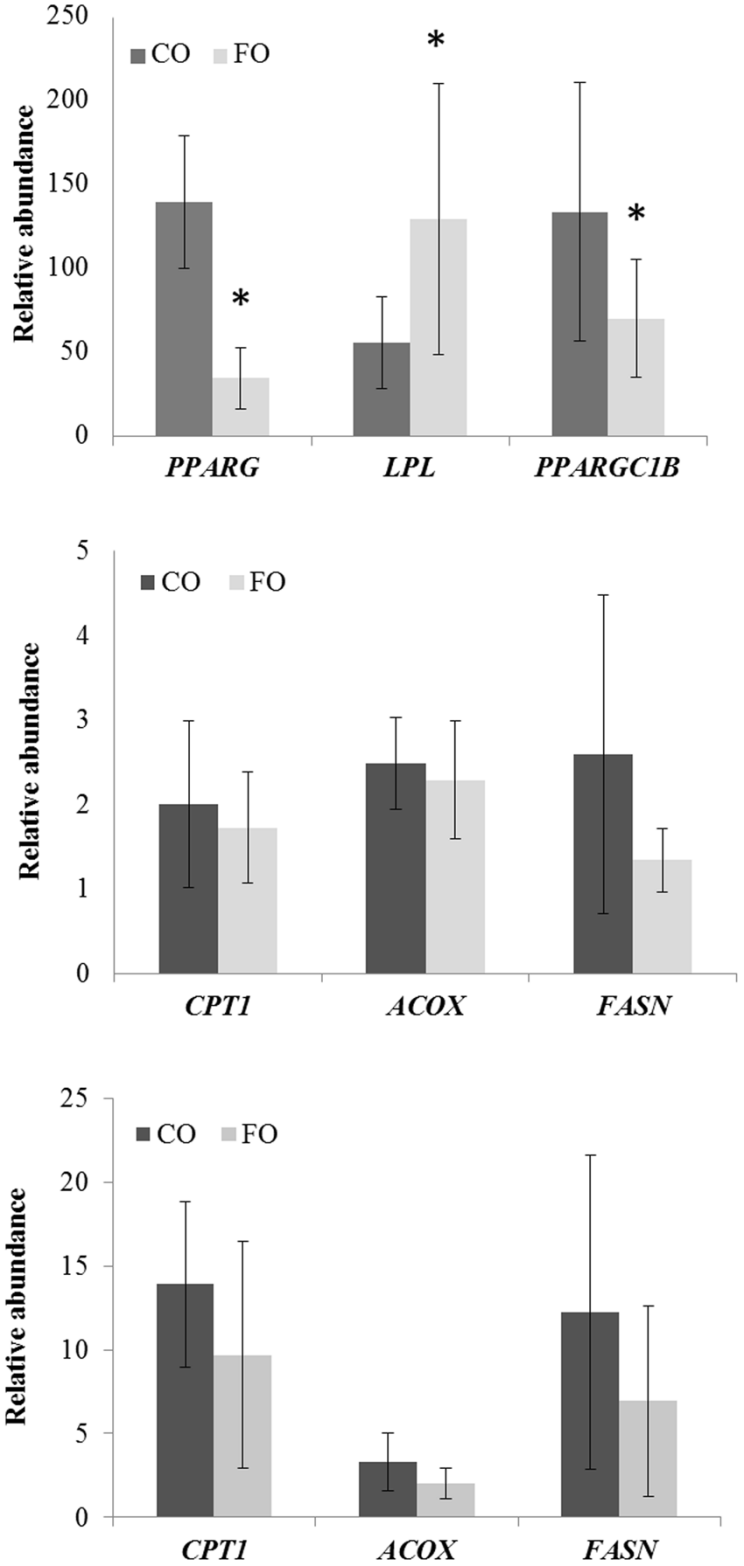
Expression of genes involved in adipogenesis, fatty acid oxidation and uptake of fatty acids in abdominal adipose tissue and liver of CO and FO chicks at 14 d. Expression of each gene of interest in adipose tissue (**A**,**B**) and liver (**C**) was normalized to that of *TBC1D8*, used a reference gene. Data shown are average relative expression values, ± SD, N = 5–6/diet group. Expression levels between CO and FO for each gene were compared using T-test. *Different from CO, *P* < 0.05. *ACOX1*, Acyl-CoA oxidase 1; *CPT1*, Carnitine palmitoyltransferase 1; *PPARG*, Peroxisome proliferator-activated receptor gamma; *PPARGC1B*, Peroxisome proliferator-activated receptor gamma co-activator 1 Beta; *FASN*, Fatty acid synthase; *LPL*, Lipoprotein lipase; CO, corn oil; FO, fish oil.

Potential mechanisms for the difference in adiposity were evaluated based on expression of genes that mediate fatty acid metabolism and adipogenesis. As shown in Fig. 3A, adipose tissue from FO chicks expressed significantly lower levels of peroxisome proliferator activated receptor gamma (*PPARG*) and its coactivator PPARGC1B (PPARG coactivator 1 β) than tissue from CO chicks (*P* < 0.05). Conversely, expression of lipoprotein lipase (*LPL*) was approximately 2.2-fold higher in adipose tissue from FO vs. CO (*P* < 0.05). Expression of carnitine palmitoyltransferase 1 (*CPT1*; mitochondrial fatty acid oxidation), acyl-coenzyme A oxidase 1 (*ACOX1*; peroxisomal fatty acid oxidation), and fatty acid synthase (*FASN*; de novo lipogenesis) did not differ significantly between groups (Fig. 3B; *P* > 0.05). Liver is the primary site of de novo lipogenesis in avians (as in humans)^24^ and plays an important role in fat deposition in broiler chickens^25^. Diet did not significantly affect expression of *CPT1*, *ACOX1* and *FASN* in liver (Fig. 3C), consistent with comparable hepatic triglyceride content in FO and CO chicks (data not shown).

### Adipose proteomics

The proteomes of adipose tissue from FO and CO chicks were analyzed by LC-MS/MS and compared to identify additional pathways that were altered by maternal FO feeding. A total of 95 known proteins differed significantly (*P* < 0.05) between FO and CO chicks (Table 4). Functional enrichment analysis revealed that this set of proteins was enriched (adj. *P* < 0.05) for components of cytoskeletal organization and cellular tight junctions, and for proteins involved in glycolysis and gluconeogenesis (Table 5). Proteins associated with the cytoskeleton (e.g., actins, vimentin (VIM), tubulins) were more abundant in FO vs. CO adipose tissue. Maternal FO also increased levels of the adipocyte lipid droplet protein perilipin (PLIN1) and Ras-related protein Rab-18 (RAB18; both ~ 1.9-fold, FO/CO), although these changes did not meet the criterion for statistical significance (*P* = 0.051 and 0.053, respectively). Glycolytic proteins were downregulated by maternal FO. Fructose-1, 6-bisphosphatase 1 and 2 (FBP1 and FBP2), pyruvate kinase (PK), enolase 3 (ENO3), and phosphoenolpyruvate carboxykinase 2 (PCK2) were significantly less abundant in tissue of FO vs. CO chicks. Two fatty acid binding proteins, fatty acid binding protein1 (FABP1) and liver basic fatty acid binding protein (LBFABP), were also present at significantly lower levels in FO adipose tissue. Functional enrichment indicated that proteins involved in muscle development and filament structure differed between FO and CO chicks.

**Table 4:** Differentially expressed proteins in abdominal adipose tissue of FO vs. CO chicks at 14 d.

	FO/CO ratio	P-value^2^	Protein symbol	Protein name
Increased (FO/CO)	4.20	0.017	EPB41L1	erythrocyte membrane protein band 4.1-like 1
	3.49	0.002	VIM	vimentin
	3.24	0.023	HBAA	hemoglobin alpha, subunit A
	3.04	0.000	TBA1	tubulin alpha-1 chain
	3.01	0.024	ANK1	ankyrin 1, erythrocytic
	2.97	0.034	NFASC	neurofascin
	2.71	0.001	DST	dystonin
	2.69	0.001	FMO3	dimethylaniline monooxygenase [N-oxide-forming]
	2.69	0.000	ACTG1	actin, gamma 1
	2.65	0.000	ACTB	actin, beta
	2.64	0.001	TBB7	tubulin beta-7 chain
	2.63	0.000	ACT5	actin, cytoplasmic type 5
	2.62	0.042	COL28A1	collagen, type XXVIII, alpha 1
	2.55	0.022	SPTB	spectrin, beta, erythrocytic
	2.55	0.001	ALB	albumin
	2.49	0.011	A2ML2	alpha-2-macroglobulin-like 2
	2.44	0.012	ITIH2	inter-alpha-trypsin inhibitor heavy chain 2
	2.40	0.046	IGF2R	insulin like growth factor 2 receptor
	2.29	0.028	BDH1	3-hydroxybutyrate dehydrogenase, type 1
	2.16	0.035	FBLN1	fibulin 1
	2.12	0.008	TUBA3	tubulin alpha-3 chain-like
	2.01	0.042	PELO	pelota homolog
	2.00	0.051	PLIN1^*^	perilipin 1
	1.98	0.054	RAB18^*^	ras-related protein Rab-18
	1.88	0.017	RBP4	retinol-binding protein 4
	1.85	0.017	ANXA5	annexin A5
	1.83	0.003	TMED7	transmembrane emp24 protein transport domain containing 7
	1.79	0.003	TF	transferrin
	1.78	0.054	VDAC3	voltage dependent anion channel 3
	1.70	0.024	TUBAL3	tubulin alpha like 3
	1.69	0.002	TUBB4B	tubulin beta 4B class IVb
	1.69	0.007	TUBA3E	tubulin alpha 3e
	1.65	0.010	TUBA4B	tubulin alpha 4b
	1.63	0.024	PLA2G6	phospholipase A2 group VI
	1.61	0.034	NSF	N-ethylmaleimide sensitive factor, vesicle fusing ATPase
	1.57	0.032	ERAP1	endoplasmic reticulum aminopeptidase 1
	1.54	0.034	DNM1L	dynamin 1 like
	1.53	0.003	TBB3	tubulin beta-3 chain
	1.52	0.007	USP9X	ubiquitin specific peptidase 9, X-linked
	1.50	0.043	PIT54	PIT54 protein
Decreased				
(FO/CO)	0.56	0.020	TXN	thioredoxin
	0.51	0.046	HMGCL	3-hydroxymethyl-3-methylglutaryl-CoA lyase
	0.45	0.019	PKM	pyruvate kinase, muscle
	0.44	0.031	TNX	avian tenascin X
	0.41	0.045	LAMA5	laminin, alpha 5
	0.38	0.024	PPA2	pyrophosphatase
	0.33	0.008	FXR1	fragile X mental retardation, autosomal homolog 1
	0.33	0.034	GIF	gastric intrinsic factor
	0.31	0.033	NT5C2	5′-nucleotidase, cytosolic II
	0.31	0.033	NT5C3	cytosolic purine 5′-nucleotidase
	0.31	0.014	FBP1	fructose-1,6-bisphosphatase 1
	0.28	0.050	UQCRFS1	ubiquinol-cytochrome c reductase, Rieske iron-sulfur polypeptide 1
	0.25	0.039	LBFABP	liver basic fatty acid binding protein
	0.22	0.032	ATP2A3	ATPase, Ca++ transporting, ubiquitous
	0.21	0.008	MYH1G	myosin, heavy chain 1 G, skeletal muscle
	0.20	0.009	CKM	creatine kinase, muscle
	0.20	0.009	MYH1C	myosin, heavy chain 1 C, skeletal muscle
	0.20	0.006	MYL1	myosin, light chain 1, alkali; skeletal, fast
	0.19	0.021	PCK2	phosphoenolpyruvate carboxykinase 2
	0.18	0.004	MYH1B	myosin, heavy chain 1B, skeletal muscle
	0.17	0.003	MYH1F	myosin, heavy chain 1 F, skeletal muscle
	0.17	0.020	MYL3	myosin, light chain 3, alkali; ventricular, skeletal, slow
	0.17	0.006	CRYAB	crystallin, alpha B
	0.17	0.005	MYSS	myosin heavy chain, skeletal muscle
	0.15	0.005	MYH1E	myosin, heavy chain 1E, skeletal muscle
	0.15	0.003	MYH1D	myosin, heavy chain 1D, skeletal muscle
	0.15	0.021	PDLIM5	PDZ and LIM domain 5
	0.15	0.004	ENO3	enolase 3
	0.15	0.002	MYLPF	myosin light chain, phosphorylatable, fast skeletal muscle
	0.15	0.013	AK1	adenylate kinase 1
	0.15	0.002	MYH1A	myosin, heavy chain 1 A, skeletal muscle
	0.14	0.007	DMD	dystrophin
	0.13	0.002	LAMA2	laminin, alpha 2
	0.13	0.003	GATM	glycine amidinotransferase
	0.13	0.002	FABP1	fatty acid binding protein 1
	0.12	0.000	ANKRD2	ankyrin repeat domain 2
	0.11	0.003	MYH13	myosin, heavy chain 13, skeletal muscle
	0.11	0.004	MYH15	myosin, heavy chain 15
	0.10	0.000	MYL2	myosin, light chain 2, regulatory, cardiac, slow
	0.10	0.003	MYSC	myosin heavy chain, cardiac muscle isoform
	0.10	0.000	ADPRHL1	ADP-ribosylhydrolase like 1
	0.10	0.007	MYOZ2	myozenin 2
	0.10	0.000	FBP2	fructose-1,6-bisphosphatase 2
	0.09	0.027	SRCA	sarcalumenin
	0.09	0.002	XIRP1	xin actin-binding repeat containing 1
	0.09	0.002	ATP2A1	ATPase, Ca++ transporting, cardiac muscle, fast twitch 1
	0.08	0.000	TMOD4	tropomodulin 4
	0.08	0.022	TNNI2	troponin I type 2
	0.08	0.001	AMPD1	adenosine monophosphate deaminase 1
	0.06	0.052	MYOM1	myomesin 1
	0.06	0.020	APOBEC2	apolipoprotein B mRNA editing enzyme, catalytic polypeptide-like 2
	0.05	0.010	CAMSAP1	calmodulin regulated spectrin associated protein 1
	0.04	0.003	TNNT3	troponin T type 3
	0.04	0.004	CASQ2	calsequestrin 2
	0.04	0.009	MYOZ1	myozenin 1

FO/CO ratio is the ratio of normalized protein abundance in abdominal adipose tissue at 14 d from N = 3 samples per diet group; CO, corn oil; FO, fish oil. P-values are from T-tests, FO vs. CO. *Included to show trend because of potential biological relevance.

**Table 5:** Functional enrichment of differentially abundant proteins in adipose tissue of chicks produced from hens fed diets containing CO or FO for 28 d.

GO Enrichment	Term	Count	Adj. *P*-value
ID			
GO:0007010	Cytoskeleton organization	11	1.16 × 10^−6^
GO:0030049	Muscle filament sliding	7	3.85 × 10^−6^
GO:0006094	Gluconeogenesis	5	3.49 × 10^−3^
GO:0007517	Muscle organ development	6	3.31 × 10^−3^
GO:0007017	Microtubule-based process	4	3.26 × 10^−2^
GO:0006928	Movement of cell or subcellular component	5	3.26 × 10^−2^
KEGG Pathway			
**ID**	**Pathway**	**Count**	**Adj. P-value**
04530	Tight junction	7	0.0052
00010	Glycolysis/gluconeogenesis	5	0.0297

The set of differentially abundant proteins in adipose tissue at 14 d was functionally annotated using the Functional Annotation and Pathway Mapping options in DAVID (v 6.8). ID refers to the specific GO term or KEGG pathway. Count represents the number of differentially abundant proteins that are annotated with each GO term or are components of each KEGG pathway; CO, corn oil; FO, fish oil. Adj. *P*-value represents the *P*-value for overrepresentation of GO terms or KEGG pathways after adjusting for false discovery using Bonferroni correction.

## Discussion

The very early onset of obesity in children highlights the need to understand how the maternal diet and lifestyle influence adipose accumulation after birth. This study fills a gap in knowledge by demonstrating that the types of fatty acids supplied to the developing embryo before birth influence adipose development. More specifically, enriching the maternal diet in FO reduced chick adiposity when compared to a maternal diet based on CO. All chicks were fed a CO-based diet after hatch, restricting the dietary manipulation to the period prior to hatch. The feeding protocol that we used was developed to enrich eggs for the consumer market in DHA and EPA. Although we did not measure yolk fatty acids, we did find marked enrichment of EPA and DHA in liver and brain phospholipids at hatch, indicating diet enriched the developing embryo as expected.

Attempts to retrospectively link fatty acid content of the maternal diet to child adiposity in humans have been inconclusive^26^. These types of studies are limited by reliance on BMI, which is a coarse index of adiposity in children, and on dietary recall to assess fatty acid intake. However, recent prospective studies using more sensitive measures of body composition and of fatty acid status have demonstrated an inverse relationship between maternal AA relative to EPA and DHA (AA/DHA + EPA) and childhood adiposity. A study of 227 mother-child pairs revealed that AA/DHA + EPA in maternal circulation mid-pregnancy predicted adiposity in children at three years of age^27^. In a much larger study 4,830 mother-child pairs, mid-pregnancy levels of EPA, DHA and docosapentaenoic acid (22:5 n-3) were associated with lower percentages of body fat and abdominal fat in children at a median age of six years^20^. Likewise, maternal levels of n-6 PUFA were associated with increased childhood adiposity. The ratio of AA/DHA + EPA in transitional breast milk, which reflect dietary patterns in the previous 90 days, was also recently shown to predict body fat percentage at four months of age^28^. Interestingly, none of these studies found a significant relationship with body weight or BMI, just as we found no effect of maternal FO feeding on chick body weight or growth, suggesting specific effects on adipose tissue. Each of these studies profiled fatty acids in the maternal blood, which provides a sensitive assessment of intake during pregnancy. However those levels may not completely reflect fatty acid delivery to the embryo, which also depends upon transfer across the placenta^29^. They also may be confounded by the child’s dietary intake after birth and lactation, particularly those that measured adiposity a few years after birth. Our findings complement these studies by demonstrating that fatty acids provided during embryonic development alone are sufficient to alter adiposity.

The shift towards increased small adipocytes coupled with downregulated expression of *PPARG* and its coactivator *PPARGC1B* suggest that maternal FO reduced adiposity in part by inhibiting progression through adipogenesis. EPA and DHA can act as ligands to activate PPARG, which would be expected to promote adipogenesis through this nuclear receptor’s role in orchestrating adipocyte differentiation. However, *in vitro* studies have shown both pro-and anti-adipogenic effects of EPA and DHA, which may be due to variation in cell lines, differentiation protocols, reference treatments, and fatty acid concentrations^30–32^. Interestingly, a shift towards increased frequency of small adipocytes and increased expression of *PPARG* has been described in fat-1 mice, which endogenously synthesize n-3 PUFA due to transgenic expression of a novel fatty acid desaturase from *C. elegans*^33^. Microarray data indicated that constitutive synthesis of n-3 PUFAs within adipocytes of fat-1 mice markedly suppressed expression of GATA binding protein 3, which normally inhibits the progression of preadipocytes into differentiation by directly suppressing PPARG^34^.

Reduced availability of glycerol-3-phosphate and fatty acids for triacylglycerol synthesis may have restricted hypertrophy of FO adipocytes. Esterification of fatty acids into triacylglycerol requires a steady supply of glycerol-3-phosphate. In adipose tissue, this is synthesized during glycolytic metabolism of glucose, and from pyruvate through glyceroneogenesis^35,36^. Levels of several glycolytic proteins and of PEPCK, which is rate-limiting for glyceroneogenesis, were reduced in FO vs. CO adipose tissue. In chickens (and humans) the majority of stored fatty acids are delivered to adipose tissue from the liver. Lipoprotein lipase, which cleaves fatty acids from circulating lipoproteins, was upregulated in FO adipose tissue. In combination, these effects suggest that maternal FO may have reduced adipocyte size, at least in part, by attenuating the capacity to esterify and store fatty acids as triacylglycerol, despite the increased fatty acid extraction that could be expected from increased LPL expression. Whether this is a primary effect of maternal FO or a secondary response to reduced delivery of fatty acids from liver cannot be determined, as we did not measure plasma VLDL levels. However, hepatic lipogenesis (based on expression of FASN) and triglyceride content (data not shown) did not differ between FO and CO, suggesting that diet did not alter the supply of fatty acids from liver.

A growing body of literature illustrates that the structural assembly of lipid droplets influences lipid metabolism in adipocytes. Maternal FO feeding increased the abundances of three proteins, PLIN1, VIM and RAB18, which localize to the surface of adipocyte lipid droplets and play key roles in balancing lipid storage and mobilization. Perilipin1 is the major surface protein of adipocyte lipid droplets, where it orchestrates lipolysis by controlling access of lipase enzymes to triacylglycerol molecules^37–39^. Vimentin is an intermediate filament that scaffolds lipid droplets to maintain their individual structural integrity^40^. Ras-related protein Rab-18 facilitates exchange of lipids between lipid droplets and the endoplasmic reticulum, playing roles in both lipolysis and lipogenesis^41,42^. All three proteins are critical for the physical remodeling and trafficking that are necessary for storage and mobilization of lipids. The physiological significance of increased levels of VIM, PLIN1 and RAB18 in FO adipose tissue requires further study, but the roles of each protein and their interactions in lipid mobilization suggests that this response may facilitate lipid utilization within adipocytes. Consistent with this possibility, overexpression of PLIN1 was shown to induce fatty acid oxidation in white adipocytes^43^. Follow-on studies are prompted to determine how maternal FO influences adipocyte architecture and if this contributes to the reduction in adiposity.

The mechanisms through which maternal consumption of LC n-3 PUFA can reduce adiposity in offspring remain to be determined. Transcriptional control through PPARs by EPA, DHA and their metabolites would require sustained enrichment of those fatty acids within adipose tissue. Fatty acid profiling demonstrated that the total lipid pool of FO chicks was enriched in both EPA and DHA up to 14 d, although the fold-enrichment relative to the CO group decreased between weeks one and two. How long this enrichment persists, especially when the post-hatch diet is not supplemented with FO, remains to be determined. Epigenetic modifications of genes involved in adipose deposition may also underlie reduced adiposity in FO chicks. In a recent randomized, controlled clinical study, fish oil supplementation during pregnancy was shown to differentially methylate 21 chromosomal regions at birth, with some differences persisting to five years of age^44^. Circulating DHA levels, both early in pregnancy and at birth, were significantly correlated with methylation of PPAR-α in infants^45^, indicating the potential for epigenetic programming of lipid metabolism. Interestingly, abundance of Apolipoprotein B mRNA editing enzyme catalytic subunit 2 (APOBEC2), was markedly reduced (~ 16-fold) in FO vs. CO adipose tissue, according to our proteomics data. This enzyme is part of a coordinated DNA demethylase system that regulates cell fate in the developing embryo through epigenetic modification^46^. Expression of APOBEC2 is primarily associated with skeletal muscle, in which it is linked to myoblast differentiation, but it is also expressed in adipose tissue in chickens^47^. If maternal FO feeding reduced adiposity through epigenetic mechanisms, which are often stable it has important implications for new means to control childhood (and potentially adult) obesity. Follow-on studies to characterize methylation patterns and other epigenomic marks of maternal FO feeding are needed to explore this possibility.

Chickens provide a unique means to address maternal programming by dietary fatty acids, but several caveats and limitations should be noted. Fish oil and CO were the primary sources of dietary fatty acids in our model. This was intentional, to maximize enrichment of the yolk in EPA and DHA. However, fatty acid consumption in human diets is more diverse and complex, and the level of enrichment we achieved may not occur in the context of a typical human diet. Nevertheless, our results provide proof-of-principle that fatty acids provided prior to birth regulate adipose development. As such, they contribute additional rationale for further studies in humans to determine if maternal fatty acid intake can be used to reduce the risk of childhood obesity. Intake of most fish species during pregnancy is now encouraged by the U.S. Food and Drug Administration, easing previous concerns about safety of fish oil intake through foods^48^. Our study was also limited to measuring adiposity within a short period (up to 14 days) after hatch, so the longevity of reduced fatness is unknown. Recent studies linking maternal levels of EPA and DHA during pregnancy with child fatness at up to six years of age encourage the interpretation that the programming effects of FO may persist. It should also be noted that, although our focus herein was on adipose tissue, it is reasonable to expect that development of other tissues could have been affected by maternal FO consumption. This is particularly possible for bone, muscle and cartilage, which arise from the same progenitor cell population as adipocytes. Further studies with in depth characterization of body composition will be necessary to explore potential additional effects of maternal fish oil consumption on development.

Similarities and differences in metabolism between chickens and humans should also be noted. In both species, liver is the primary site of de novo lipogenesis^49,50^, which contrasts with rodents and swine^51,52^. Transcriptomic studies of chicken adipose tissue in several labs, including ours, have shown that many of the genes involved in fat deposition in humans are differentially expressed between lean and fat lines of chickens^53–56^. For example, genes involved in adipogenesis, de novo lipogenesis, and triglyceride synthesis are upregulated in chickens with relatively high levels of adiposity. The various lean and fat model pairs used in these studies arose from different methods (e.g., phenotypic selection for abdominal fat^54^, growth rate^55^, feed efficiency^56^, and use in production^53^), indicating the robustness of these gene-phenotype relationships. A notable metabolic difference between chickens and humans is that chickens are naturally hyperglycemic compared to mammals, although regulation of insulin release and pancreatic β cell function are similar^57–59^. Insulin signaling is attenuated in skeletal muscle and adipose tissue (but not liver^60,61^). Accordingly, chickens are considered as a model of early insulin resistance that may be relevant for studies of diabetes in humans^61^.An ortholog of the leptin gene was not identified in the first few annotations of the chicken genome, which raised questions about potential differences in adipose biology between the two species^62^. Recently, however, improved annotation and sequencing of GC-rich regions led to characterization of chicken leptin, as well as orthologs of other mammalian genes that are expressed in adipose tissue^63,64^.

In summary, our data demonstrate that maternal fish oil consumption reduces adipose deposition in offspring. Our study was limited to the first two weeks of life, and follow-on experiments are necessary to determine how long this effect persists as chicks mature. These results complement recent studies in humans that link LC n-3 PUFA in the maternal diet to reduced adipose mass in children. Accordingly, they highlight the potential to attenuate fat accumulation and potentially the risk for childhood obesity through dietary intervention prior to birth.

## Methods

### Diets and husbandry

Animal husbandry procedures were reviewed and approved by the Institutional Animal Care and Use Committees of the University of Georgia (broiler breeder hens) and the University of Tennessee (chicks), and all methods were performed according to relevant guidelines and regulations of these two institutions. Cobb 500 broiler breeder hens were maintained at the University of Georgia. For 28 d, hens (N = 30/diet) were fed broiler breeder diets that were supplemented with either CO (Conagra Brands; Chicago IL) or FO (Jedwards International, Braintree MA). This specific source of FO was produced from anchovies and contained 18% EPA and 12% DHA. The two diets were prepared by adding either CO or FO (2.3% wt:wt) to a commercially-formulated broiler-breeder diet consisting primarily of corn and soybean meal. Formulas for each diet are shown in Supplemental Table 2. The final fat content of the two diets was 5.8%, with 49.7% (CO) and 43.6% (FO) from n-6 of fatty acids, and 2.2% (CO) and 5.2% (FO) from n-3 fatty acids. Fatty acid composition of each diet is shown in Supplemental Table 3. After feeding hens for 28 d, fertilized eggs were collected from each hen and transported to the University of Tennessee for incubation and hatching. Multiple roosters were used for fertilization of eggs in each diet group. Eggs were weighed and incubated for three weeks, until hatch. Hatch rates were calculated for each group as a percentage of eggs that produced viable chicks. At hatch, chicks were grouped by hen diet (CO or FO) and housed separately in brooder cages (n = 10/cage) at standard brooding temperature 35 °C. Each cage was equipped with a feeder and drinker to which birds had ad libitum access. All chicks (both CO and FO) were fed a standard broiler starter diet in which added fat (3% fat wt:wt) was supplied from CO, using the same source of oil that was used to prepare the hen CO diet. Weight gain and feed intake were monitored weekly.

### Blood and tissue collection

Chicks were euthanized by CO_2_ asphyxiation. Two chicks from each group were euthanized at hatch for collection of liver and brain for lipid analyses. Samples of each tissue were snap-frozen and stored at −80 °C. The remaining chicks were euthanized at 7 and 14 d of age. At the time of euthanasia blood was collected by cardiac venipuncture and transferred to 10 ml serum separator tubes (Fisher Scientific, Pittsburgh, PA). Serum was separated by centrifugation and stored at −80 °C until analyses of circulating metabolites. Abdominal and femoral (subcutaneous) adipose depots were dissected and weighed as indices of adiposity. Samples of each depot and of liver were subsequently snap-frozen in liquid nitrogen and stored at −80 °C. Samples of abdominal adipose tissue were fixed for 24 h at 4 °C in paraformaldehyde (4%) for determination of adipocyte size by histology.

### Serum metabolites

Commercially available colorimetric assay kits were used to measure serum glucose (Cayman Chemical, Ann Arbor, MI) and non-esterified fatty acid (NEFA) levels (Wako Chemicals, Neuss, Germany).

### Fatty acid analysis

Abdominal fat samples from five randomly selected birds in each diet and age were analyzed for fatty acid composition by GC with flame ionization detection (GC-FID) by the W.M. Keck Metabolomics Research Laboratory (Iowa State University, Des Moines, IA). Tissues were pulverized under liquid nitrogen using a stainless steel mortar and pestle. Approximately 100 mg of pulverized tissue was weighed from each bird and extracted in glass vials in chloroform:methanol (2:1). Extracted fats were trans-esterified with sodium methoxide. The fatty acid methyl esters (FAMES) were extracted into hexane and analyzed using an Agilent 7890 A GC-FID, with Agilent CP-Wax 52CB column (15 m, 0.32mm, 0.5um; Agilent, Santa Clara, CA). The oven starting temperature of 100 °C, increased to 170 °C with a ramp of 2 °C/min, increased to 180 °C with a ramp of 0.5°C/min, to a final temperature of 250°C with a ramp of 10°C/min. A mix of FAME standards (Supelco 37 FAME mix; catalog # CRM47885 SUPELCO, Bellefonte, PA) was used to generate a calibration curve for identification and quantification of FAMES. Data for each fatty acid were expressed as mole% ± SEM. The activities of stearoyl-CoA desaturase (SCD) and delta-6 desaturase (D6D), and indices of de novo lipogenesis (DNL) and elongation (EL) were estimated from mole% values using the following equations: 1.) SCD-16: 16:1n-7/16:0; 2.) SCD-18: 18:1n-9/18:0; 3.) D6D: 18:3n-6/18:2n-6; 4.) DNL: 16:0/18:2n-6; 5.) EL: 18:0/16.

### Phospholipid analysis

Fatty acid composition of phosphatidylcholine species in brain and liver collected at hatch (n = 2/diet) and in abdominal adipose tissue collected at 7 d of age (n = 5/diet) was analyzed using UltraHigh Performance Liquid Chromatograph (UHPLC)-MS (Thermo-Fisher Scientific, Waltham, MA). Tissue samples (100 mg) were pulverized under liquid nitrogen using a mortar and pestle. Phospholipids were extracted using a modified Bligh and Dyer protocol^65^. Dried extracts were resuspended in 300μL of methanol/chloroform (9:1) for UHPLC-MS analysis as previously described^66^. Lipid species were identified using exact *m/z* and retention times. Lipid standards (Avanti Polar Lipids, Alabaster AL) from each phospholipid class were run to verify retention times. All ion fragmentation was used to confirm that phosphatidylcholines contained DHA and EPA as acyl chains. For all ion fragmentation scans, the resolution was 140,000 with a scan range of 100-1500 *m/z*. The normalized collision energy was 30 eV with a stepped collision energy of 50%. Lipids were identified by their fragments using Xcalibur software (Thermo Fisher Scientific, San Jose, CA). Data analysis was performed using Maven software^67^.

### Adipocyte size

Abdominal fat samples from three birds per diet at each of the two ages were embedded, sectioned and stained with hematoxylin and eosin (H&E; two slides/bird) for determination of adipocyte size, as previously described by^53^. The three birds with adiposity values closest to the median adiposity within each diet/age group were used. Briefly, images of three independent fields per slide were captured on each slide under 20x magnification with the Advanced Microscopy Group EVOS XL Core microscope (Fisher Scientific, Pittsburgh, PA). For consistency, the same person performed all measurements. Image J (Version 1.48, National Institutes of Health) was used to determine adipocyte area, (μm^2^), using microscope settings of 2.8 μm/pixel, and using the restriction that measurements must exceed 500 μm^2^. Frequency distributions were produced by grouping adipocytes into bins based on area and counting the frequency of cells within each bin. A standard method was used to calculate adipocyte volume and number^68^.

### Real time PCR assay

Total RNA was isolated from approximately 200 mg of abdominal adipose tissue and liver from five chicks at 14 d in each diet group using Invitrogen^TM^ TRIzol^TM^ (Invitrogen, Carlsbad, CA). The five birds with adiposity values closest to the median adiposity within each diet were used. CDNA was synthesized from 500 ng total RNA in 20 μl reactions using iScript cDNA Synthesis kit (Bio-Rad Laboratories, Hercules, CA). Predesigned and validated primers for quantitative real-time PCR (QPCR) were purchased from Qiagen (Quantitect; Germantown, MD). QPCR was performed in triplicate for each sample using iQ SYBR Green Master Mix (Bio-Rad Laboratories, Hercules, CA), as previously described^53^. Expression levels of genes of interest were normalized to expression of TBC1 domain family, member 8 (*TBC1D8*) used as a housekeeper.

### Proteomics

Approximately one g of abdominal adipose tissue from each of three chicks per diet was pulverized in liquid nitrogen, of which approximately 60 mg was used for protein extraction. Proteins were extracted using a detergent-free, methanol/chloroform (2:1) protein extraction protocol^69^ designed for lipid-rich tissues and based on the Bligh and Dyer method^70^. Proteins were precipitated from the aqueous fraction using trichloroacetic acid and digested with sequencing grade trypsin. Approximately two mg of proteolytic peptides were obtained from each sample after clean up. Fifty µg aliquots of these peptides were used for 2D-LC-MS/MS proteomic measurements on an LTQ Orbitrap mass spectrometer (Thermo Fisher Scientific, Waltham, MA), as previously described^71^. MyriMatch v2.1.111^72^ was used to search the raw mass spectra against the predicted protein database to identify fully-tryptic peptides, which were then grouped together into respective proteins with IDPicker v.3^73^. Only protein identifications with at least two identified peptide spectra and a maximum q-value of 0.02 were considered for further analysis. Peptide fragments were mapped to proteins in the *Gallus gallus* genome (V3.0) using Uniprot.

### Statistical Analysis

Statistical analyses were performed using SAS (V 9.4). Data were checked for normality using Shapiro-Wilks prior to statistical testing. Body and adipose weights, serum metabolites, tissue fatty acid composition, average adipocyte size and adipocyte number were analyzed using mixed model ANOVA with terms for diet, age and their interaction (diet X age). Significant F-tests (P < 0.05) were followed by post-hoc testing using least square means (LSM) to identify pairwise differences between groups. The frequencies of adipocytes within each bin at each age, as well as QPCR data, were compared between CO and FO using independent t-tests. Proteins that differed in abundance between CO and FO adipose tissue were also identified by t-test after protein spectral counts were log-transformed and pareto-scaled in MetaboAnalyst 3.0^74^ to normalize data distributions. Functional analysis of differentially abundant proteins was performed using the Functional Annotation and Pathway Mapping options found in the Database for Annotation, Visualization and Integrated Discovery (DAVID, V 6.8)^75^. All statistical tests were performed using *P*-values ≤ 0.05 as the criterion for statistical significance.

### Data availability

The datasets generated during the current study are available from the corresponding author on reasonable request

## Electronic supplementary material

Supplemental information and data
