# Author Correction: Maternal consumption of fish oil programs reduced adiposity in broiler chicks

**DOI:** 10.1038/s41598-018-29512-5

**Published:** 2018-07-27

**Authors:** Ronique C. Beckford, Sarah J. Howard, Suchita Das, Abigail T. Farmer, Shawn R. Campagna, Jiali Yu, Robert L. Hettich, Jeanna L. Wilson, Brynn H. Voy

**Affiliations:** 1 Department of Animal Science, University of Tennessee, Knoxville, TN United States; 2 Department of Chemistry, University of Tennessee, Knoxville, TN United States; 3 Graduate School of Genome Science and Technology, University of Tennessee, Knoxville, TN United States; 4 Chemical Sciences Division, Oak Ridge National Laboratory, Oak Ridge, TN United States; 5 Department of Poultry Science, University of Georgia, Athens, GA United States

Correction to: *Scientific Reports* 10.1038/s41598-017-13519-5, published online 13 October 2017

The original version of this Article contained an error in Figure 3A that resulted in the values for corn oil (CO) and fish oil (FO) being switched.

As a result, in the Abstract,

“This adipocyte profile was paralleled by upregulated expression of the adipogenic regulator *PPARG* and its co-activator *PPARGC1B*, and reduced expression of *LPL*.”

now reads:

“This adipocyte profile was paralleled by lower expression of the adipogenic regulator *PPARG* and its co-activator *PPARGC1B*, and elevated expression of *LPL*.”

In the Results section, under the subheading ‘QPRC’,

“As shown in Fig. 3A, adipose tissue from FO chicks expressed significantly higher levels of peroxisome proliferator activated receptor gamma (*PPARG*; 4.4-fold) and its coactivator *PPARGC1B* (PPARG coactivator 1 β; 3.4-fold) than tissue from CO chicks (P < 0.05). Conversely, expression of lipoprotein lipase (LPL) was approximately 60% lower in adipose tissue from FO vs. CO (P < 0.05).”

now reads:

“As shown in Fig. 3A, adipose tissue from FO chicks expressed significantly lower levels of peroxisome proliferator activated receptor gamma (*PPARG*) and its coactivator *PPARGC1B* (PPARG coactivator 1 β) than tissue from CO chicks (P < 0.05). Conversely, expression of lipoprotein lipase (LPL) was approximately 2.2-fold higher in adipose tissue from FO vs. CO (P < 0.05).”

In the Discussion,

“The shift towards increased small adipocytes coupled with upregulated expression of *PPARG* and its coactivator *PPARGC1B* suggest that maternal FO promoted adipogenesis, despite reducing adiposity.”

now reads:

“The shift towards increased small adipocytes coupled with downregulated expression of *PPARG* and its coactivator *PPARGC1B* suggest that maternal FO reduced adiposity in part by inhibiting progression through adipogenesis.”

Additionally, in the Discussion,

“Lipoprotein lipase, which cleaves fatty acids from circulating lipoproteins, was also down-regulated in FO adipose tissue. In combination, these effects suggest that maternal FO may have reduced adipocyte size, at least in part, by attenuating the capacity to extract, esterify and store fatty acids as triacylglycerol.”

now reads:

“Lipoprotein lipase, which cleaves fatty acids from circulating lipoproteins, was upregulated in FO adipose tissue. In combination, these effects suggest that maternal FO may have reduced adipocyte size, at least in part, by attenuating the capacity to esterify and store fatty acids as triacylglycerol, despite the increased fatty acid extraction that could be expected from increased LPL expression.”

These errors have now been corrected in the HTML and PDF versions of this Article.

