# Supplementary material for: Maternal consumption of fish oil programs reduced adiposity in broiler chicks
Source: Sci Rep. 2017 Oct 13;7:13129. doi: 10.1038/s41598-017-13519-5 (PMC5640664; doi:10.1038/s41598-017-13519-5)
Supplement: Supplementary file 1 — Supplemental information and data [file 41598_2017_13519_MOESM1_ESM.pdf]

Supplementary data

Maternal consumption of fish oil programs reduced adiposity in broiler chicks

Ronique C. Beckford, Sarah J. Howard, Suchita Das, Abigail T. Farmer, Shawn R. Campagna, Jiali Yu, Robert L. Hettich, Jeanna L. Wilson, Brynn H. Voy

Supplemental table 1. Phosphatidylcholine (PC) species containing EPA and DHA in abdominal adipose tissue of FO vs. CO chicks at 7d

	% Total		
PC species	CO	FO	P-value
PC(38:7)	0.06±0.01	0.22±0.09	0.03
PC(38:6)	0.71±0.09	2.85±1.29	0.03
PC(40:9)	0.01±0.00	0.03±0.01	0.02
PC(40:8)	0.09±0.01	0.29±0.16	0.07
PC(40:7)	0.24±0.04	0.81±0.40	0.05
PC(40:6)	0.64±0.12	2.44±0.94	0.02
PC(42:8)	0.05±0.01	0.13±0.07	0.09
PC(42:7)	0.06±0.02	0.18±0.08	0.05
PC(42:6)	0.09±0.03	0.18±0.07	0.09
PC(44:8)	0.02±0.01	0.04±0.03	0.26
PC(44:7)	0.02±0.01	0.04±0.02	0.19
PC(44:6)	0.03±0.01	0.05±0.02	0.13

% total is the percentage of total PC species detected in abdominal adipose tissue at 7 d from N=5 samples per diet group; values are means ± SD; CO, corn oil; FO, fish oil
P-value from T-test, FO vs. CO;

Supplemental table 2. Formulation of the FO and CO diets

Ingredient	Amount (%)	
	CO	FO
Corn	63.2	63.2
Soybean meal (48 %)	11.9	11.9
Wheat midds	8.00	8.00
Limestone	7.90	7.90
Pro-pak[1]	5.00	5.00
Fish oil	-	2.30
Corn oil	2.30	-
Mono calcium phosphate	0.60	0.60
Termin-8[2]	0.30	0.30
Salt	0.20	0.20
Vit Pre-mix[3]	0.20	0.20
Bicarbonate of soda	0.15	0.15
DL-Methionine	0.14	0.14
Choline	0.12	0.12
TM Pre-mix[4]	0.08	0.08
	Calculated nutrient analysis	
ME, kcal/kg	2938	2938
CP, %	15.2	15.2
Calcium, %	3.50	3.50
Total phosphorus	0.61	0.61
Available phosphorus	0.43	0.43

CO, corn oil; FO, fish oil

[1]Protein concentrate, H.J. Baker and Bros., 595 Summer Street, Stamford, CT 06901-1407

[2]Antimicrobial preservative

[3]Vitamin mix provided per kilogram of complete diet: vitamin A, 30,800 IU; Vitamin D_3, 9,250 IU; vitamin E, 153.9 IU; vitamin B_{12}, 0.154 mg; riboflavin, 46.2 mg; niacin, 185 mg; pantothenic acid, 84 mg; menadione sodium bisulfite, 16.2 mg; folic acid, 12.3 mg; pyridoxine HCl, 46.2 mg; thiamine HCl, 20.5 mg; biotin, 9.3 mg; choline, 2,944 mg; niacin, 185 mg

[4]Mineral mix provided per kilogram of complete diet: Cu, 55 mg; I, 7.3 mg; Fe, 366 mg; Mn, 310 mg; Zn, 321 mg; K, 2.23 g; Mg, 1.09 g; Se, 0.48 mg

Supplemental table 3. Fatty acid composition of CO and FO diets

	% of total	
Fatty acid	CO	FO
Capric, 10:0	0.28	0.21
Lauric,12:0	0.19	0.18
Myristic, 14:0	0.45	2.12
Palmitic, 16:0	15.8	18.1
Stearic, 18:0	15.2	12.8
Oleic, 18:1	10.3	11.7
Linoleic, 18:2n-6 *cis*	25.9	22.5
Linoleadic, 18:2n-6t *trans*	23.6	20.5
α-Linolenic, 18:3n-3	1.7	0.85
γ-Linolenic, 18:3n-6	0.15	0.32
Eicosadienoic, 20:2n-6	0.04	0.07
Dihomo- γ-Linolenic, 20:3n-6	0.03	0.05
Eicosatrienoic 20:3n-3	0.08	0.12
Arachidonic, 20:4n-6	0.02	0.04
Eicosapentaenoic, 20:5n-3	0.15	2.2
Docosadienoic, 22:2n-6	0.02	0.10
Docosahexaenoic, 22:6n-3	0.29	2.0
Total n-3	2.2	5.2
Total n-6	49.7	43.6
Total fat	5.8	5.8

% of total is the relative percentage of fatty acid methyl esters measured in each sample; CO, corn oil; FO, fish oil. Total n-3 is the sum of C18:3, 20:3, 20:5 and 22:6 n-3 fatty acids. Total n-6 is the sum of C18:2, 18:3, 20:2, 20:3, C20:4 and 22:2 n-6 fatty acids.
Total fat is the % of fat (wt:wt) in the diet.
